# Chemical Studies of Multicomponent Kidney Stones Using the Modern Advanced Research Methods

**DOI:** 10.3390/molecules28166089

**Published:** 2023-08-16

**Authors:** Weronika Sofińska-Chmiel, Marta Goliszek, Marek Drewniak, Aldona Nowicka, Marcin Kuśmierz, Agnieszka Adamczuk, Paulina Malinowska, Ryszard Maciejewski, Małgorzata Tatarczak-Michalewska, Eliza Blicharska

**Affiliations:** 1 Analytical Laboratory, Institute of Chemical Sciences, Faculty of Chemistry, Maria Curie Skłodowska University, Maria Curie Skłodowska Sq. 2, 20-031 Lublin, Poland; 2 Institute of Agrophysics Polish Academy of Sciences, Doświadczalna 4 Str., 20-290 Lublin, Poland; 3 Department of Anatomy, Medical University of Lublin, Jaczewskiego 4 Str., 20-090 Lublin, Poland; 4 Institute of Health Sciences, The John Paul II Catholic University of Lublin, Kostantynów 1 H Str., 20-708 Lublin, Poland; 5 Department of Pathobiochemistry and Interdisciplinary Applications of Ion Chromatography, Medical University of Lublin, 1 Chodźki Str., 20-093 Lublin, Poland

**Keywords:** kidney stones, scanning electron microscopy, X-ray diffraction, X-ray photoelectron spectroscopy, infrared spectroscopy, FTIR microscopy

## Abstract

Defining the kidney stone composition is important for determining a treatment plan, understanding etiology and preventing recurrence of nephrolithiasis, which is considered as a common, civilization disease and a serious worldwide medical problem. The aim of this study was to investigate the morphology and chemical composition of multicomponent kidney stones. The identification methods such as infrared spectroscopy (FTIR), X-ray diffraction (XRD), and electron microscopy with the EDX detector were presented. The studies by the X-ray photoelectron spectroscopy (XPS) were also carried out for better understanding of their chemical structure. The chemical mapping by the FTIR microscopy was performed to show the distribution of individual chemical compounds that constitute the building blocks of kidney stones. The use of modern research methods with a particular emphasis on the spectroscopic methods allowed for a thorough examination of the subject of nephrolithiasis.

## 1. Introduction

Nephrolithiasis is a common, civilization disease which is now a serious medical problem [1]. There are many differences in the prevalence rate of kidney stone worldwide, so this rate is reported as 1–5% in Asia, 5–9% in Europe, and 7–15% in North America [2]. In the last three decades, the prevalence of kidney stones has increased worldwide [3,4,5,6]. Possible explanations for this growing trend include the obesity epidemic, higher prevalence of gout and diabetes, and poor diet [5,7]. The increased prevalence of nephrolithiasis is followed by a significantly higher financial burden for healthcare systems [8]. This is a recurrent disease—the probability of recurrence within 5 years is estimated to be 50% [9,10]. Its main cause is the deposition of organic compounds and minerals in the parts of the excretory system such as the kidneys, ureters, and bladder. This process is induced by different factors such as the genetic predisposition, environmental conditions or dietary habits [11]. A high-protein diet and low fluid intake throughout the day have a significant effect on urine thickening and crystallization of kidney stone crystals in the tubules [12]. The causes of kidney stones are also related to the electrolyte disturbances [13,14]. The literature reports show that obesity, diabetes and hypertension also increase the risk of developing kidney stones [15,16]. The process of crystallization is complex and not fully understood. There are four stages of this process: nucleation, growth, aggregation of the crystal and retention of the crystals in the kidney, i.e., formation of deposits [17]. The numerous types of kidney stones result from the varying chemical compositions of the stones, which also vary in frequency of occurrence in the urinary tract [18]. Globally, approximately 80% of kidney stones are composed of calcium oxalate mixed with calcium phosphate. Stones composed of uric acid, struvite and cystine are also common and account for approximately 9%, 10% and 1% of stones, respectively [1,19]. With the help of modern analytical techniques, it is possible to get to know their exact structure and chemical composition in order to develop treatment, prophylaxis or prevention of recurrence of this disease.

Kidney stone disease is frequent in high-income countries, and in the United States and Europe, the prevalence reaches up to 10%. In addition, it shows high rates of recurrences. Swift changes in the global population and socio-economic and climate conditions are expected to transform the map of kidney stones epidemiology in the years to come worldwide. Of consequence, primary and secondary prevention of kidney stone disease is a relevant task [6]. Defining the stone composition is important for determining a treatment plan, understanding etiology and preventing recurrence [20]. The studies of the phase composition of kidney stones are very important to elucidate their etiology. Research on kidney stones can be performed using many different techniques, from simple observations using stereoscopic microscopy to complex techniques, such as scanning electron microscopy with energy-dispersive X-ray microanalysis (SEM–EDS) and many others. The literature reports various methods, among which the most common are: Fourier transform infrared spectroscopy (FTIR) and scanning electron microscopy (SEM) [21], X-ray fluorescence (XRF) and X-ray diffraction (XRD) which are very often regarded as relevant methods to make the phase analysis of this type of materials [22,23] and finally, the XPS photoelectron spectroscopy, which is one of the latest methods of analysing kidney stones, enabling the determination of the thorough elemental composition and determination of the characteristic chemical bonds present in a given compound [24]. There are a few reports in the literature on the use of the Rietveld method for the quantitative phase analysis and microstructural characterization of phases to identify kidney stones [25]. The limitations in the application of this method are crystallographic heterogeneity and different degrees of crystallinity of this type of materials [26,27].

Costa-Bauzá et al. [28] used in their studies stereoscopic microscopy together with scanning electron microscopy, elemental microanalysis by EDS and infrared spectroscopy. The authors observed that although the major components of a kidney stone can be obtained directly by IR spectroscopy which is the most commonly used analytical method, but IR spectroscopy data alone are no longer sufficient to determine the etiology of different types of renal stones. Only a detailed structural study and an analysis of the major and minor components obtained by combination of the methods mentioned above are needed to provide sufficient information to determine the possible causes of stone formation.

Chatterjee et al. [26] investigated the phase composition and morphological characterization of human kidney stones using IR spectroscopy, scanning electron microscopy and X-ray Rietveld analysis and concluded that these techniques work are of practical importance for the specialists analyzing the quantitative phase composition of urinary calculi and their morphological features at the sub-micron level.

Raman spectroscopy is also a very useful method for the analysis of kidney stones. It can provide the chemical composition, structure and spatial information of the tested molecules [29].

According to literature, kidney stones are extensively studied by Raman spectroscopy [29,30,31,32]. Raman spectroscopy measures the scattering of vibrating and rotating molecules, while FTIR microscopy assesses the absorption of these molecules [33]. In general, both methods are similar and complementary. However, they have advantages and disadvantages. In our work, we decided to use FTIR spectroscopy to avoid some limitations connected to Raman spectroscopy such as possible fluorescence which can hide the Raman spectrum and sample heating through the intense laser radiation that can destroy the sample or cover the Raman spectrum.

The research presented in the paper concerns the identification of kidney stones based on the analysis of the chemical composition, structural characterization and phase quantification. The chemical composition of kidney stones was estimated by the infrared spectroscopy (FTIR) and scanning electron microscopy with the EDX detector (SEM-EDX). Using the X-ray diffraction (XRD), the phase quantification was examined. For better knowledge about the chemical structure of kidney stones, the X-ray photoelectron spectroscopy (XPS) studies were carried out. The chemical mapping by the FTIR microscopy used to show the distribution of individual chemical compounds that constitute the building blocks of kidney stones was the novelty in the research.

## 2. Results and Discussion

### 2.1. FTIR-ATR

**Figure 1:**
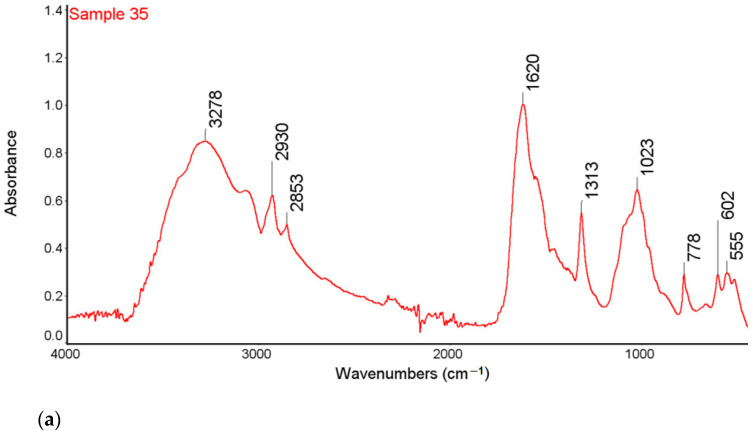

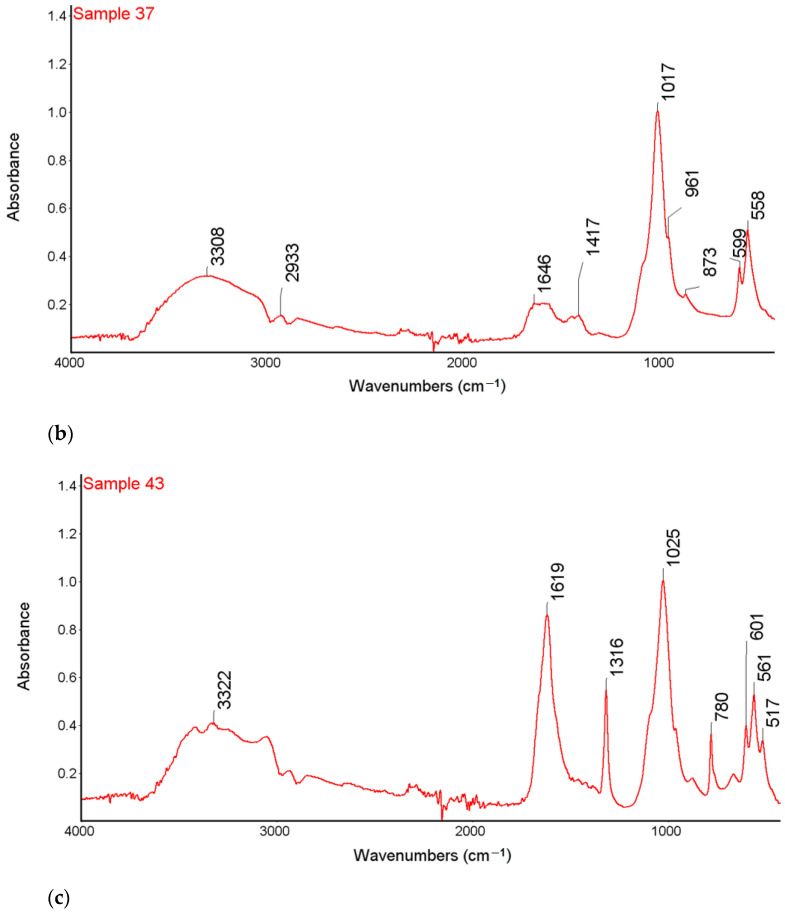
FTIR-ATR spectra of samples 35 (**a**), 37 (**b**) and 43 (**c**) with the marked spectral bands.

**Figure 2:**
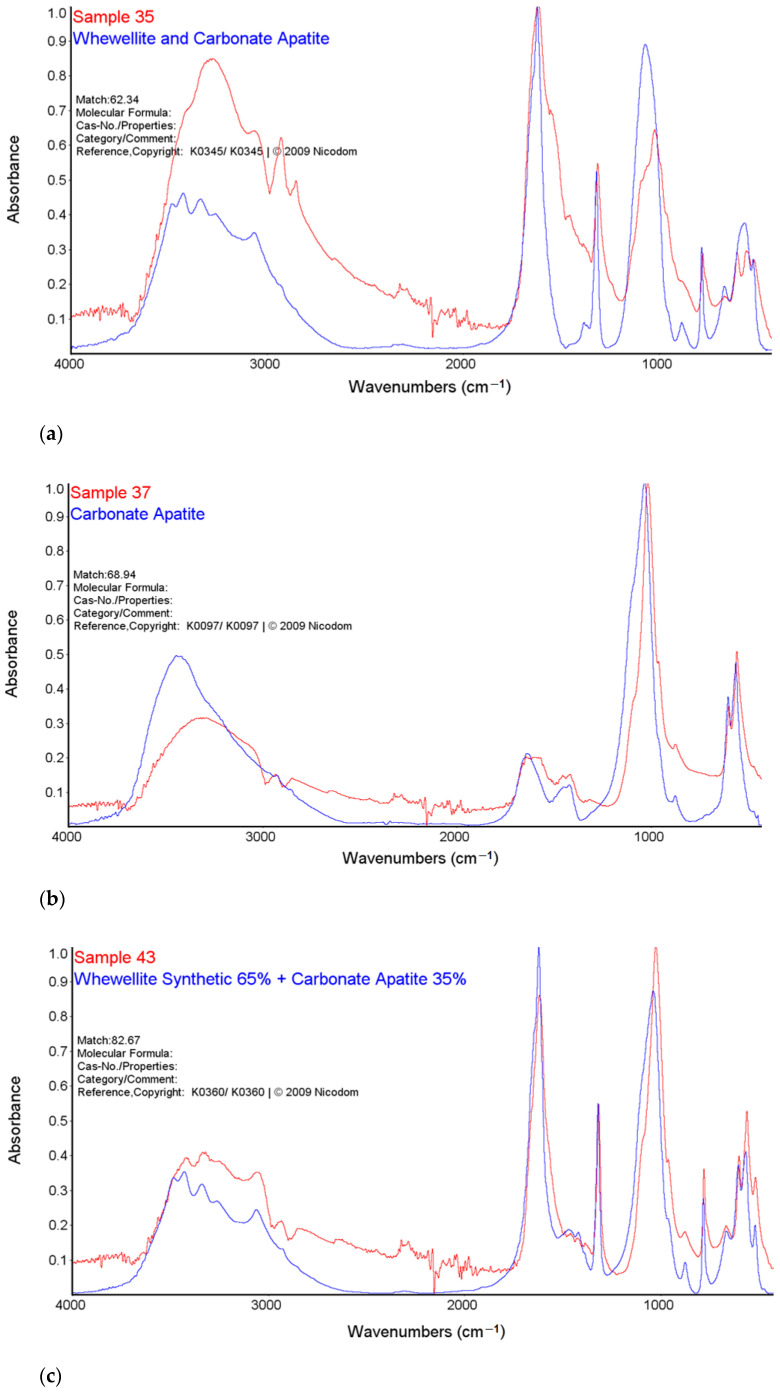
Comparison of the FTIR-ATR spectra of samples 35 (**a**), 37 (**b**) and 43 (**c**) with Nicodem Kidney Stones database.

The analysis of urinary stones is used for the diagnosis of the etiology of an episode of nephrolithiasis. The technique considered as standard for this purpose is the FTIR spectroscopy [34]. The results of the analyses are presented in Figure 1 and Figure 2.

High intensity bands in the range 3600–3200 cm^−1^ were observed in the FTIR-ATR spectra of all tested samples. This band is characteristic of the vibration stretching of the O-H and the N-H groups. In the range of 3000–2850 cm^−1^, there are bands corresponding to symmetrical and asymmetric stretching vibrations originating from the CH_2_ groups indicating the presence of organic compounds on the surface of the examined kidney stones. The research also showed the bands of low intensity, characteristic of the stretching vibrations of the P-O groups in the range of 1182–1005 cm^−1^ and the stretching vibrations of the O-P-O groups in the range of 650–500 cm^−1^ [35].

The bands at 1316 cm^−1^ correspond to the symmetrical stretching vibrations of the COO- groups and the peak at 780 cm^−1^ which corresponds to the deformation vibrations of the COO- groups from calcium oxalate of sample 35 and 43 [35,36]. The FTIR-ATR spectra showed the presence of bands characteristic of both carbonates and phosphates for sample 37 and the presence of bands characteristic of both oxalates, carbonates and phosphates for sample 43.

For the sample 35, the greatest match of the obtained spectrum is to apatite, calcium oxalate and carbonate according to the Nicodem Kidney Stones database with the coefficient of matching equals to 62.34%. The tests of sample 37 with the FTIR-ATR method showed the best match of the tested material to the mixture of carbonates and apatite. The coefficient of matching the obtained spectrum to the mentioned database was 68.94%. The FTIR-ATR tests of sample 43 showed the greatest match to calcium oxalate (65%) as well as carbonate and apatite, which together constitute 35% of the tested material. The coefficient of matching the obtained spectrum to the Nicodem Kidney Stones database is 85.99%. Using FTIR-ATR, it cannot be concluded which carbonate is present, but information from other techniques allows us to propose a tentative mineral composition for the analysed kidney stones.

**Figure 3:**
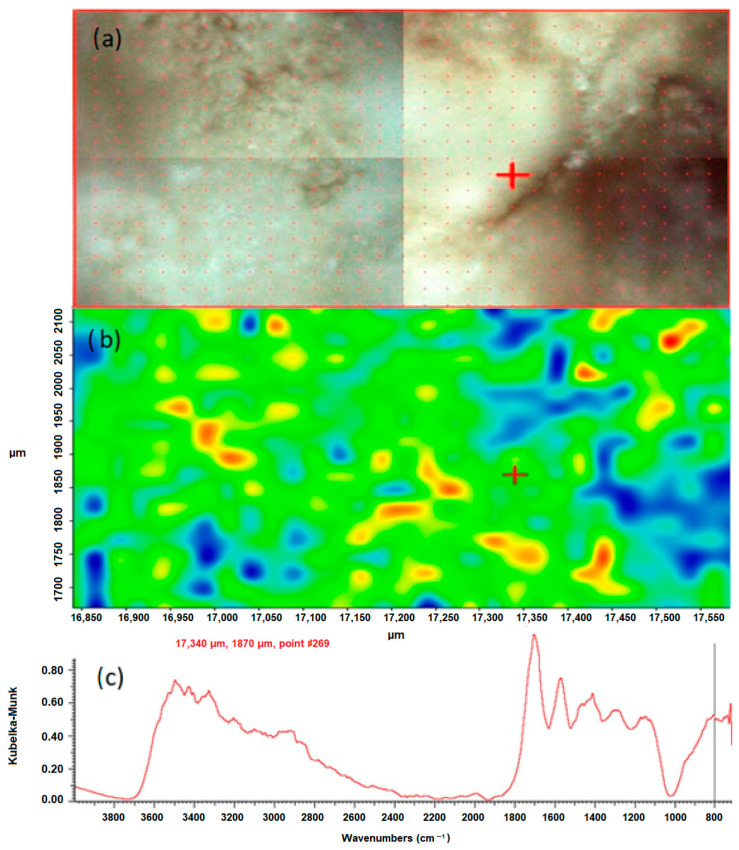
Correlation map of oxalate distribution in sample 43 after peak 780 cm^−1^, (**a**) microscopic image of the mapped area, (**b**) chemical map, (**c**) FTIR spectrum determined in the positions 17,340 µm and 1870 µm from the green area.

**Figure 4:**
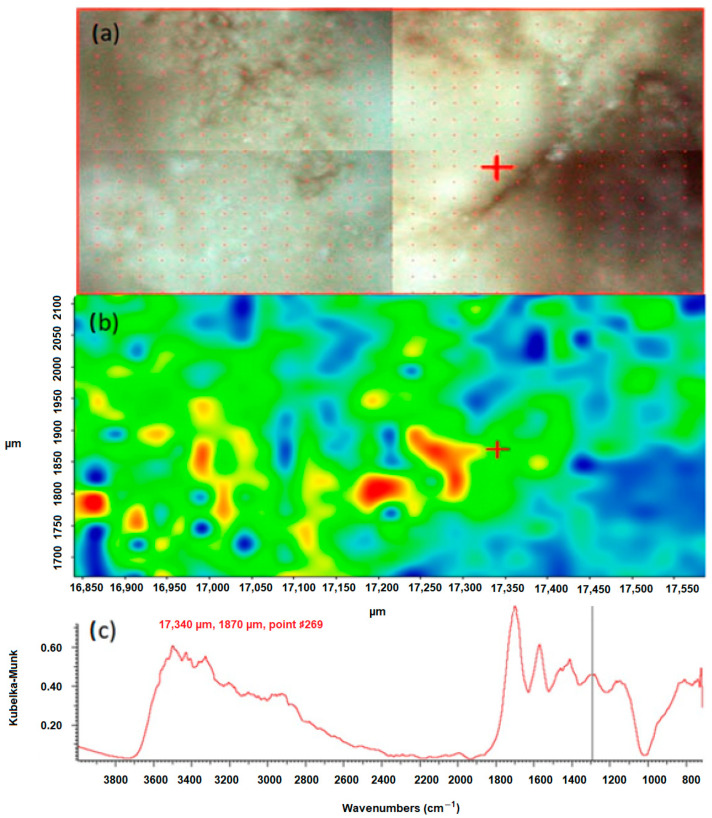
Correlation map of oxalate distribution in sample 43 after peak 1311 cm^−1^, (**a**) microscopic image of the mapped area, (**b**) chemical map, (**c**) FTIR spectrum determined in the positions 17,340 µm and 1870 µm from the green area.

The chemical mapping was performed to isolate the individual chemical compounds that constitute the components of the studied kidney stones. They were generated and processed using the Omnic Specta™ software (version 8.1) by the method of correlation to the selected band for sample 43 in the positions 780 cm^−1^ and 1311 cm^−1^. The study was carried out for the selected areas. The results are presented in Figure 3 and Figure 4.

According to the literature data, the peak position of 1311 cm^−1^ corresponds to symmetric stretching vibrations of the COO-groups. In the FTIR spectrum of the test sample 43, this peak is characteristic of calcium oxalate monohydrate and hydrated calcium oxalate, because these bonds occur only in oxalates. They do not occur in apatites, which are also a component of the sample. By creating a chemical map using the method of correlation to the peak in the position, the distribution of oxalates in the tested sample was demonstrated. In the presented maps, Figure 3b and Figure 4b red, yellow and green areas indicate where oxalates are present. The blue color indicates the distribution of apatite. An analogous map was created by correlation to the peak at 780 cm^−1^, which corresponds to the deformation vibrations of the COO- groups. The map created shows the distribution of oxalates in the kidney stone sample being tested. The red, yellow and green colors indicate the distribution of calcium oxalate monohydrate and hydrated calcium oxalate, while the blue areas correspond to the distribution of apatite.

### 2.2. X-ray Diffraction (XRD)

**Figure 5:**
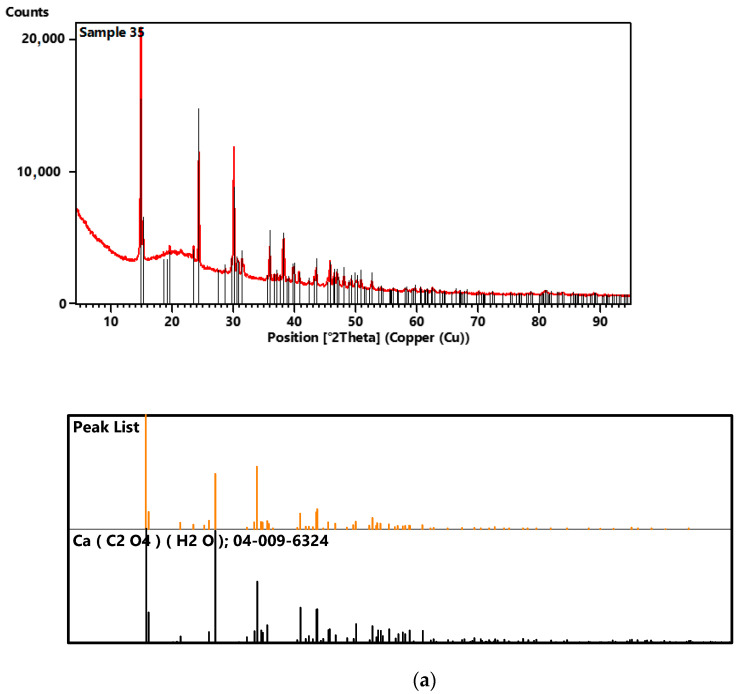

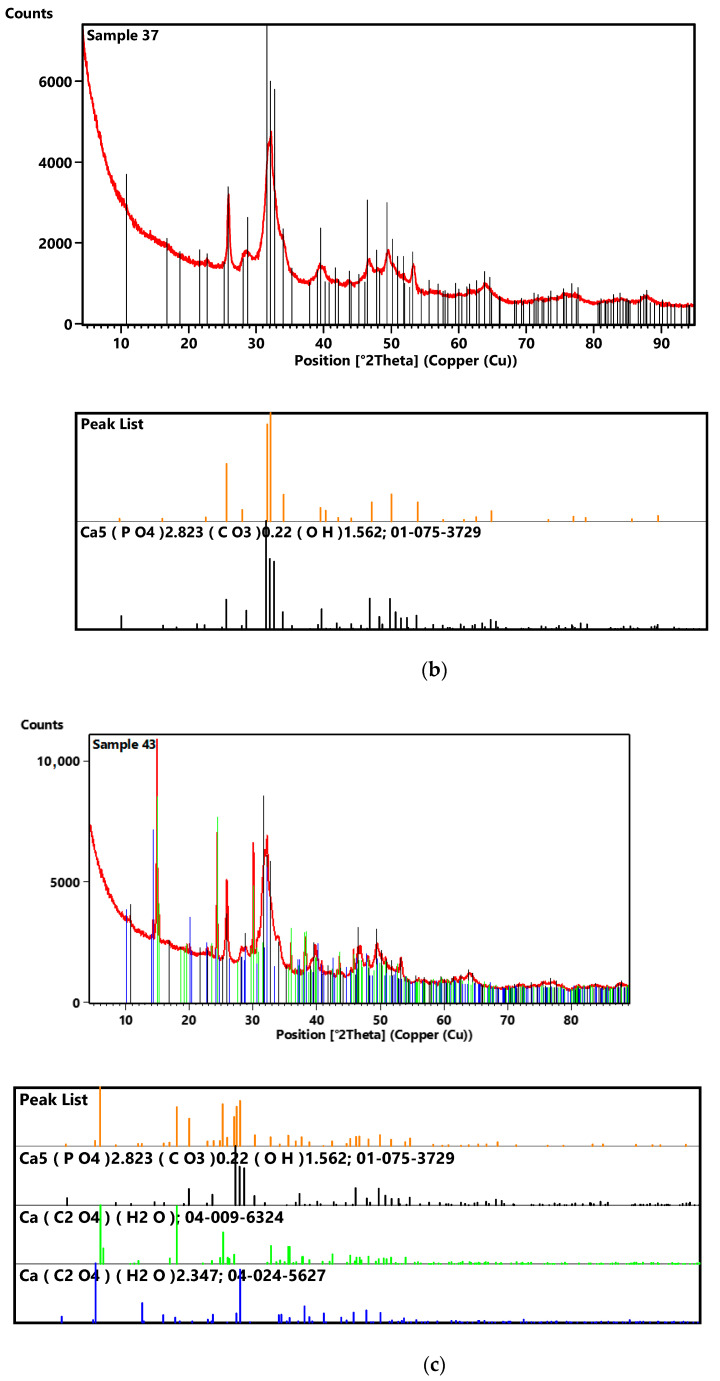
XRD diffractograms of samples 35 (**a**), 37 (**b**) and 43 (**c**).

X-ray diffraction is a versatile, non-destructive analytical technique for the identification and quantitative determination of the various crystalline forms known as phases of compounds present in powdered and solid samples. XRD works by irradiating a material with incident X-rays and then measuring the intensities and scattering angles of the X-rays that leave the material. The XRD method allows calculation of chemical composition based on the results of quantitative phase analysis. The fact that this analysis can be applied to very small samples is another advantage of the technique [22,37,38]. XRD diffractograms are presented in Figure 5.

For sample 35, the comparison of the diffraction pattern with the ICDD diffraction database PDF4+2020 showed that 100% of the crystalline phase in the test sample is calcium oxalate (Table 1).

For sample 37, the tests showed the greatest match between the tested material and calcium phosphate. Comparison of the diffractogram of sample 37 with the ICDD diffraction database PDF4+2020 showed that the main component of the crystalline phase of the test sample is calcium phosphate doped with some carbonate and hydroxyls, leading to a mixed mineral with a formula like Ca_5_[(PO_4_)_2.823_ (CO_3_)_0.22_ (OH)_1.562_].

For sample 43, the tests proved the presence of calcium oxalate monohydrate, calcium oxalate dihydrate and calcium phosphate in the test sample. Comparison of the diffractogram of sample 43 with the database shows that the main component of the test sample is calcium phosphate which represents 62% of the crystalline phase. An amount of 33% of the crystalline phase is calcium oxalate dihydrate and the remaining 5% is calcium oxalate monohydrate.

### 2.3. Scanning Electron Microscopy SEM-EDS

The advanced use of SEM–EDS in nephrology is relatively new to other analytical techniques. Scanning electron microscopy–energy-dispersive X-ray spectroscopy is a highly relevant analytical technique that reveals a detailed description of the morphology and structure of kidney stones [39].

**Figure 6:**
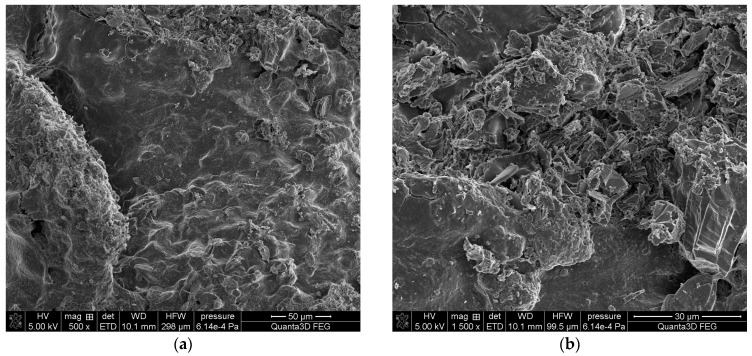
SEM images of sample 35 at magnification: (**a**) 500×, (**b**) 1500×.

**Figure 7:**
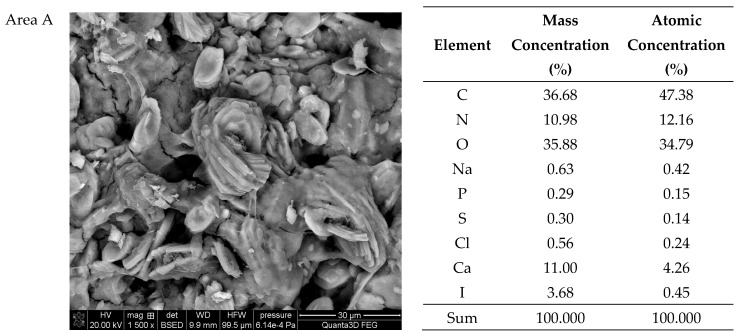

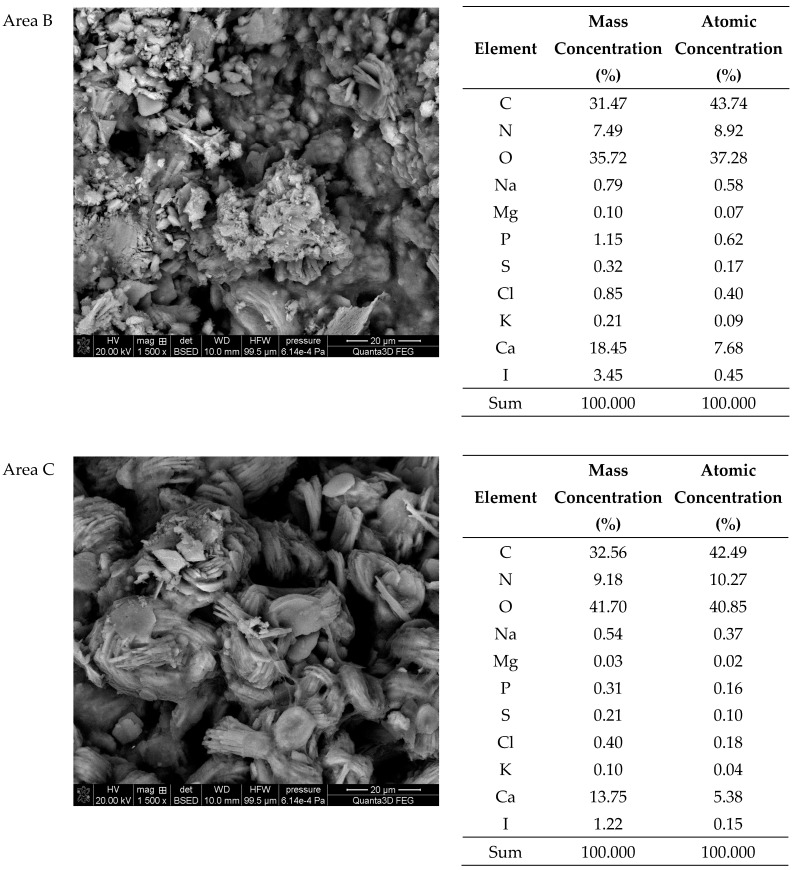
SEM images of sample 35 and the elemental composition determined by the scanning electron microscopy with the EDS detector.

The SEM-EDS tests of sample 35 (Figure 6 and Figure 7) showed a high homogeneity of the elemental composition of the tested areas A, B and C. Clear crystal structures were observed in the microscopic images. In addition to the elements included in the composition of calcium oxalate such as carbon, oxygen and calcium, the test sample also contains nitrogen, sodium, magnesium, phosphorus, sulfur, chlorine, potassium and iodine. The presence of phosphorus confirms the presence of apatite in the tested sample of kidney stones.

**Figure 8:**
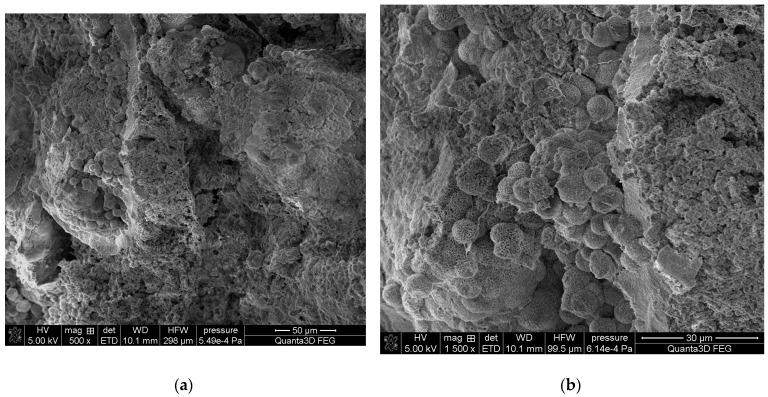
SEM images of sample 37 at magnification: (**a**) 500×, (**b**) 1500×.

**Figure 9:**
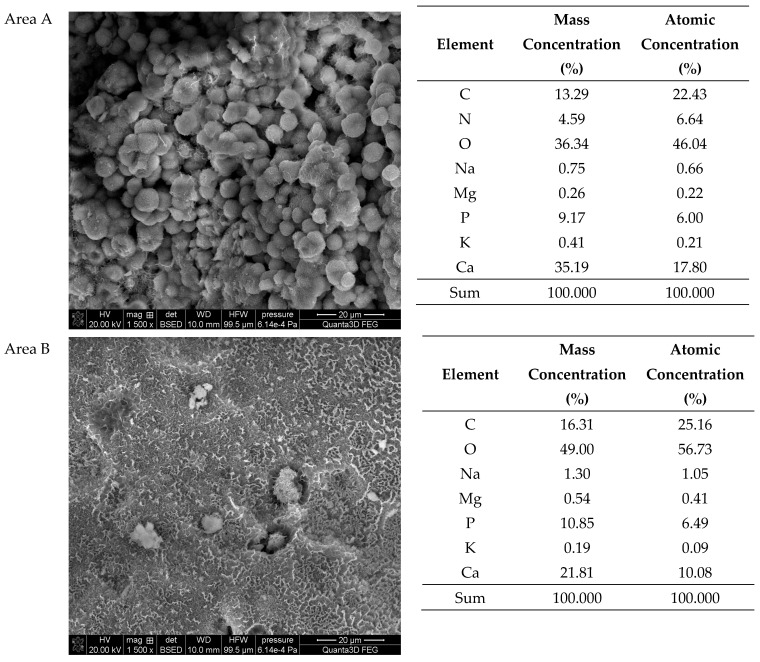

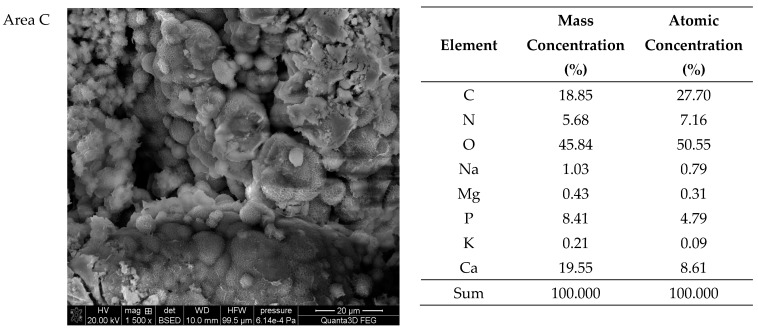
SEM images of sample 37 and the elemental composition determined by the scanning electron microscopy with the EDS detector.

The SEM-EDS studies of sample 37 (Figure 8 and Figure 9) showed also a high homogeneity of the elemental composition of the studied areas A and C. However, the composition of area B differs from the other areas in the absence of nitrogen. The microscopic images show clear crystalline structures, in the form of characteristic balls, which are most clearly visible in the areas A and C. According to the literature data, the crystalline forms from the areas A and C visible in the microscope images are characteristic of apatite [22,40]. The content of other elements in the trace amounts indicates the presence of impurities on the surface of the examined kidney stones.

**Figure 10:**
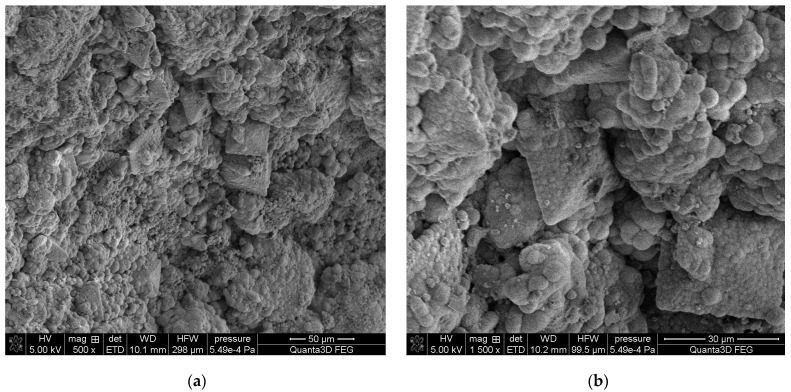
SEM images of sample 43 at magnification: (**a**) 500×, (**b**) 1500×.

**Figure 11:**
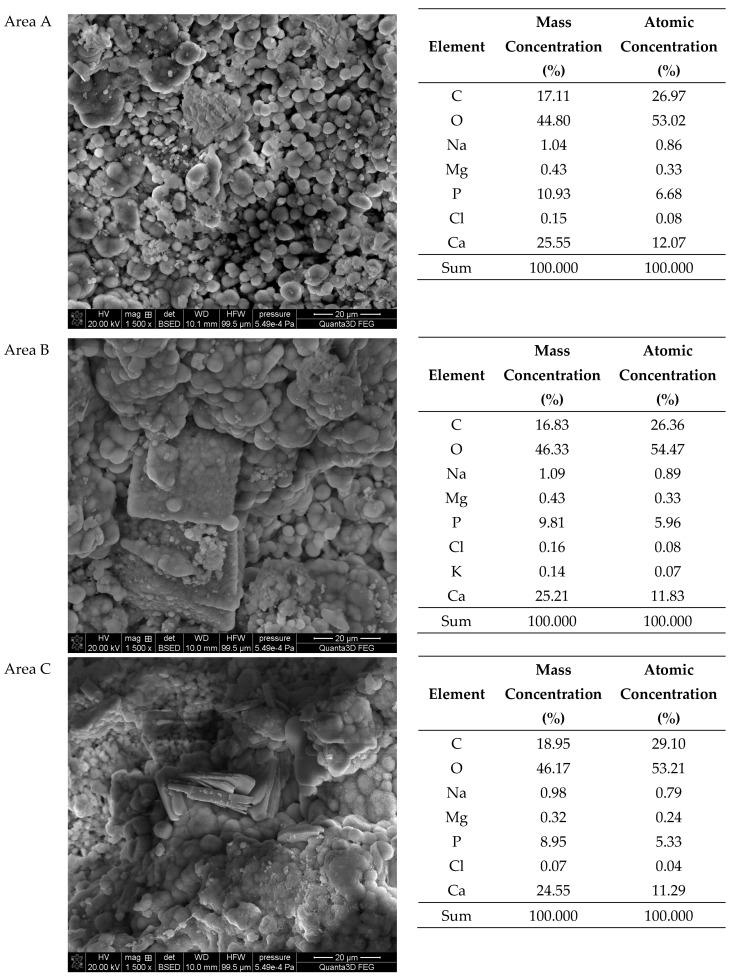
SEM images of sample 43 and the elemental composition determined by the scanning electron microscopy with the EDS detector.

The SEM-EDS studies of sample 43 (Figure 10 and Figure 11) showed a high homogeneity in the elemental composition of the areas A, B and C. Additionally, the area B of sample 43 showed the presence of potassium. The microscopic images exhibited clear structures resembling flat globules especially visible in the area A. According to the literature data, the crystalline forms visible in the microscopic images from the areas A and B (sparse in the area C) are characteristic of apatite [22,40].

The EDS-spectrograms of the tested samples were presented in the Supplementary Material (Figures S1–S3).

Due to that excess of calcium, one can conclude the generic carbonates used to explain the FTIR data are mainly calcium carbonate with minor amounts of sodium, magnesium and sometimes potassium carbonates.

### 2.4. XPS Spectroscopy

**Figure 12:**
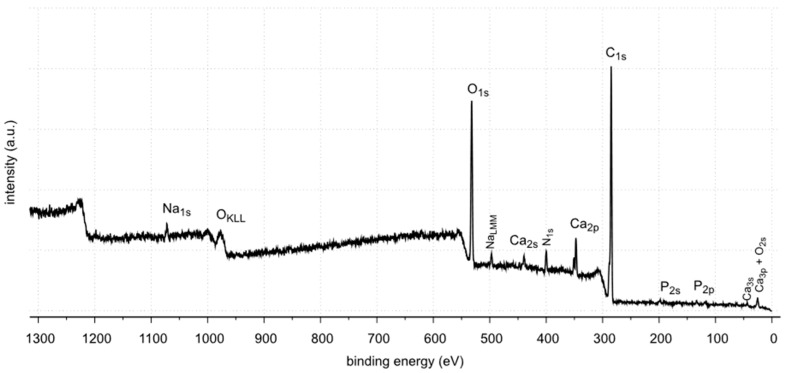
XPS survey spectrum of sample 35.

**Figure 13:**
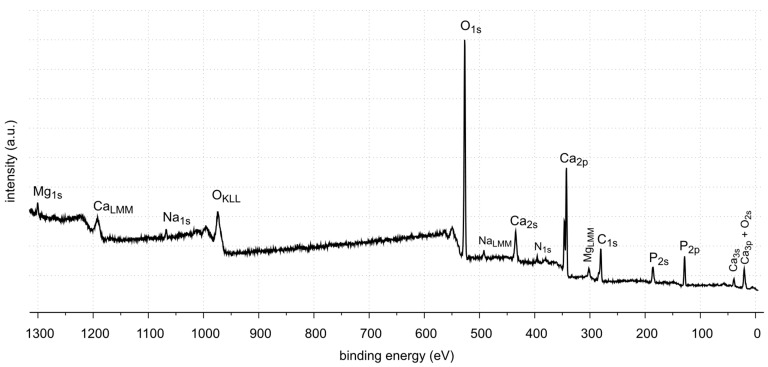
XPS survey spectrum of sample 37.

**Figure 14:**
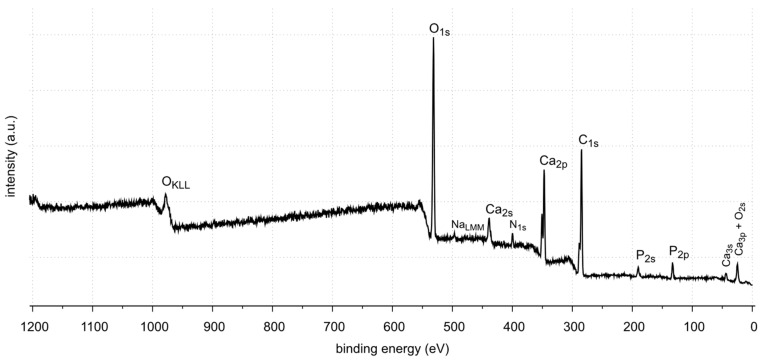
XPS survey spectrum of sample 43.

**Figure 15:**
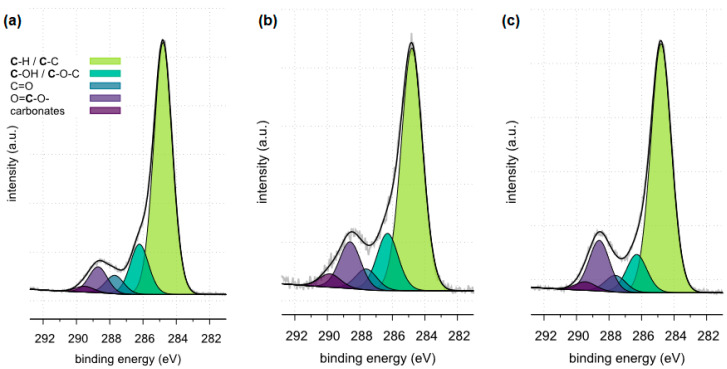
High resolution (regional) XPS spectra of carbon: (**a**) sample 35, (**b**) sample 37, (**c**) sample 43.

**Figure 16:**
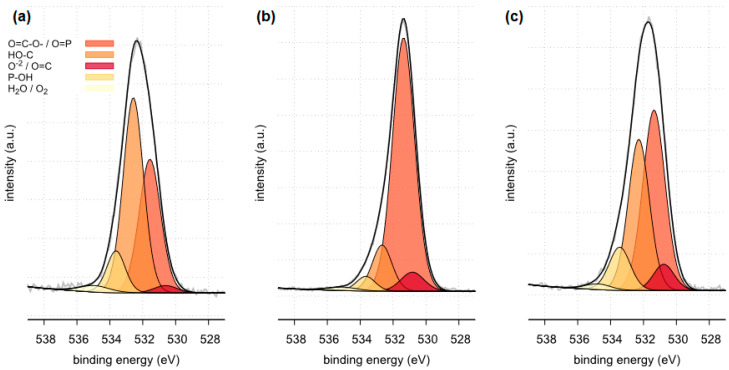
High resolution (regional) XPS spectra of oxygen: (**a**) sample 35 (**b**) sample 37, (**c**) sample 43.

**Figure 17:**
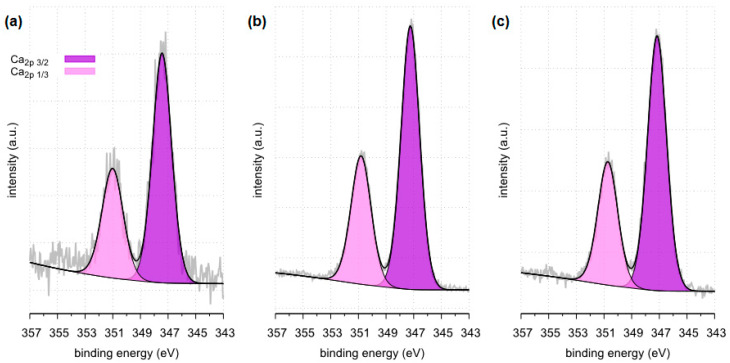
High resolution (regional) XPS spectra of calcium: (**a**) sample 35 (**b**) sample 37, (**c**) sample 43.

**Figure 18:**
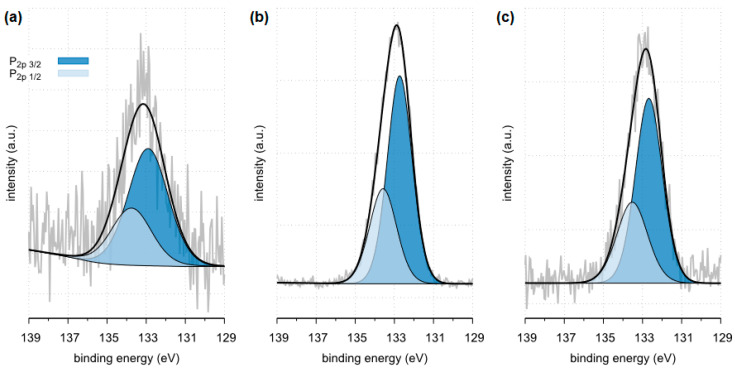
High resolution (regional) XPS spectra of phosphorus: (**a**) sample 35 (**b**) sample 37, (**c**) sample 43.

The X-ray photoelectron spectroscopy (XPS) method enables the detection and quantitative analysis of elements that are part of kidney stones (except for hydrogen and helium). This method allows for determining the type of chemical bonds in which the elements present on the surface of the tested samples are involved and to identify the chemical states of the elements (Figure 12, Figure 13, Figure 14, Figure 15, Figure 16, Figure 17 and Figure 18 and Table 2, Table 3, Table 4, Table 5, Table 6, Table 7 and Table 8).

Identification of chemical states of elements and bonds which they create may be a key aspect in understanding the chemical structure of kidney stones and the reasons for their formation.

The XPS studies of sample 35 revealed the presence of elements listed in Table 2.

XPS tests showed the presence of the following elements on the surface of the sample 35: carbon, oxygen, calcium, nitrogen, phosphorus, aluminum and sodium. Carbon, oxygen, phosphorus and calcium present on the surface are components of oxalates, carbonates and apatite, as shown by FTIR identification of sample 35. Other elements, such as aluminum and sodium, are the contaminants of the sample. Nitrogen is an element found in kidney stones in the form of uric acid. However, no uric acid was found in the tests performed. In the FTIR spectra, the C=O stretching vibration signal is not located at 1725–1700 cm^−1^.

XPS tests showed the presence of carbon, oxygen, phosphorus and calcium on the surface of sample 37. These elements are part of calcium carbonate and apatite. In addition, small amounts of magnesium and sodium were found. Nitrogen was not detected in the tested sample. Magnesium may come from magnesium carbonate, a very common component of kidney stones. However, it was not identified by XRD. This may be due to its low amount (0.7% At. Conc.) or it is in a non-crystalline form. The presence of nitrogen, as in the case of sample 35, may be related to the organic impurities in the sample.

XPS tests showed the presence of carbon, oxygen, phosphorus and calcium on the surface of sample 43. These elements are components of oxalate, calcium carbonate and apatite, which are the main components of this kidney stone sample. Tests also showed the presence of nitrogen. Looking back at FTIR-ATR analysis, as in the case of samples 35 and 37, the signal coming from the C=O stretching vibrations is not in the range of flow numbers 1725–1700 cm^−1^. Therefore, the presence of uric acid is excluded. The nitrogen probably comes from protein impurities in kidney stones.

The XPS tests carried out in a narrow range of carbon binding energy showed in all samples (samples 35, 37 and 43) the presence of carbonates, which are components of the examined kidney stones. Moreover, in the case of samples 35 and 43, the presence of C-C and C=O bonds originating from oxalates was confirmed. The presence of other carbon bonds on the surface of the tested samples is the result of organic contaminants present on the surface of the materials.

The XPS tests carried out in a narrow range of binding energies for oxygen showed for all the tested samples (samples 35, 37 and 43) the presence of carbonates, which are components of the examined kidney stones. Moreover, in the case of samples 35 and 43, the presence of C=O bonds originating from oxalate was confirmed. XPS studies in a narrow range of binding energies for oxygen also confirmed the presence of phosphates on the surface of all tested samples. The presence of P=O and P-OH bonds was confirmed in phosphates.

All tested samples showed the presence of phosphorus in the form of PO_4_^3−^ which indicates the presence of phosphates. In the case of sample 35, the presence of phosphates was confirmed by FTIR. Due to a too small amount of phosphates, they were not detected by the XRD method. XPS tests showed only 0.8 %at. conc. of this element on the surface of the tested sample. In the case of the remaining tested samples, the presence of phosphates was confirmed by both XRD and FTIR methods. The presence of phosphorus in all tested samples was also confirmed by the SEM-EDS method.

The above observations confirm the need to use various analytical methods for the proper analysis of the tested materials.

## 3. Materials and Methods

### 3.1. Research Material

**Figure 19:**
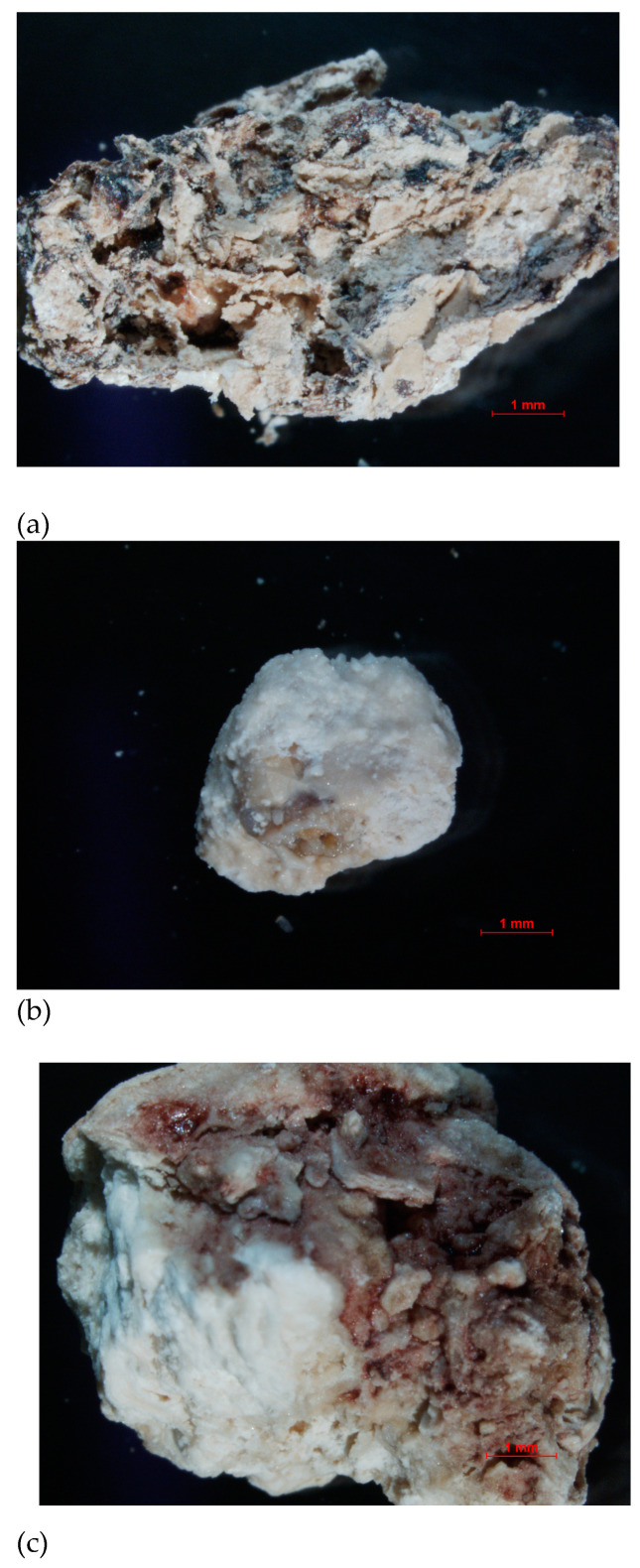
Optical microscope image of the examined samples of kidney stones: (**a**) sample 35, (**b**) sample 37, (**c**) sample 43.

The research materials were the kidney stones obtained in cooperation with the hospital laboratory and with the consent of the Bioethics Committee at the Medical University of Lublin (consent number KE-0254/140/2020). The examined kidney stones were excreted spontaneously by the patients or surgically removed in the hospital ward. The samples were washed with distilled water to remove loose debris such as blood, mucous and casts and then air-dried for several days. Stones received in sterile containers were cleaned, dried, and stored in an air-conditioned environment (between 20–22 °C) until analyzed. Stones were then pulverized with a pestle and mortar to produce a fine homogeneous powder [21]. The deposits varied in appearance, size and chemical composition. The samples were denoted with the following symbols: sample 35, 37 and sample 43 (Figure 19).

### 3.2. Methods

The FTIR-ATR spectroscopy was used for kidney stones identification. The kidney stone samples were ground in an agate mortar and analysed by the FTIR-ATR method in the powder form. A Thermo Nicolet 8700 FTIR spectrometer (Thermo Scientific, Waltham, MA, USA) with a Smart Orbit™ diamond ATR attachment and a DTGS (Deuterated Triglycine Sulphate) detector was used to record the spectra. The detector guarantees the stability of the signal in the mid-infrared spectral range: 4000–400 cm^−1^. The spectra were subjected to the operations of baseline correction, ATR correction and scaled normalization, owing to which these spectra are equivalent to the transmission spectra. The obtained spectra were compared with the commercial Nicodon Kidney Stone Database.

The chemical mapping was performed with the FTIR Nicolet iN10 MX microscope (Thermo Scientific, Waltham, MA, USA) equipped with the motorized XY stage with the use of the liquid-nitrogen-cooled matrix detector. Due to the specificity of the material, the test was carried out using a non-destructive reflection technique. Chemical maps were generated and processed using the Omnic Specta™ software (version 8.1) by correlation to the selected band.

XRD analyses were performed using the Empyrean X-ray diffractometer (Malvern PANalytical, Almelo, The Netherlands). The analysed samples were triturated in an agate mortar and the resulting powder was placed in a cuvette. Then, at a small pressure, the area of the preparation was smoothed out. The analysed samples were placed in the goniometer holder and then the measurement was performed. In this method, the location and intensity of the reflections are determined by the crystal structure model. The widths and shapes of these reflections are related by the empirical relationships. All variables e.g., lattice parameters, are adjusted in the procedure so that the diffractogram model is consistent with the experimental data. The recorded diffractograms of samples 37 and 43 were compared with the ICDD PDF4 + 2021 diffraction database.

The microscopic examinations were performed with the use of the FEI Quanta 3D FEG high-resolution scanning electron-ion microscope (Thermo Scientific, Waltham, MA, USA). The microscopic photos of the sample surface topography were taken in high vacuum (HV), in the topographic contrast mode, SE—i.e., registration of secondary electrons knocked out of the thin subsurface layer. In order to improve the contrast and reduce the loading effect, the samples were sprayed with the Pd/Au layer before the measurement by using the Polaron SC7640 sputter coater (Quorum Technologies, Lewes, UK). The photos of the samples were registered at magnifications of 500× and 1500×.

XPS analyses were made using the Ultra High Vacuum multi-chamber analytical system (Prevac) equipped with Scienta R4000 hemispherical analyser. Before the analysis, the kidney stone samples were stabletted, mounted on the molybdenum support and then degassed at room temperature to the constant high vacuum of ~5 × 10^−8^ mbar in the UHV system loading lock. After introducing the UHV system into the analysis chamber, a suitable XPS analysis was performed. A monochromatic source of AlKα radiation was used as the source of photoelectron excitation. The photoelectrons were excited by the X-ray radiation with a characteristic line AlKα and energy of 1486.7 eV generated by the VG Scienta SAX 100 lamp with an aluminum anode together with a VG Scienta XM 780 monochromator. During the collection of the XPS spectra, the samples were simultaneously irradiated with a low-energy source of electrons EFG (Electron Flood Gun (E = 2 V, Ie = 200 µA)) in order to eliminate charging. During the measurement, the vacuum in the analytical chamber was lower than 2 × 10^−8^ mbar. The CasaXPSsoftware (v 2.3.23 PR1.0) was used for data processing and calculating the results. In order to normalize the spectroscopic measurements, the X axis of the spectra (binding energy) was calibrated to the C1s aliphatic carbon peak, EB = 285 eV.

## 4. Conclusions

Kidney stones are a common civilization disease, the first mention of which appeared in ancient times. For generations, it has been a serious problem for people affected by it. Currently, owing to the progress of technology, there can be used modern analytical techniques to study kidney stones. Studies on their structure and composition enables finding new ways against this disease and preventing its recurrence. This is why the analysis and identification of kidney stones is so important. Kidney stones are a recurring disease. It is estimated that in as many as 1/4 of patients diagnosed with kidney stones, within 10 years from the diagnosis of kidney stones, further deposits are formed. In this paper, the kidney stone samples were examined using modern advanced research methods.

The use of FTIR spectroscopy with the ATR attachment allowed the identification of kidney stones. The research showed the following chemical compositions of the studied kidney stones: sample 35—oxalates, carbonates, hydroxyapatite; sample 37—carbonates, hydroxyapatite; sample 43—oxalates, carbonates, hydroxyapatite.

The chemical mapping performed by the infrared spectroscopy of the selected stone area allowed for presenting the distribution of oxalates in sample 43.

The XRD examination enabled the identification of crystalline phases in the kidney stone samples. The investigations of the elemental composition by SEM-EDX revealed the heterogeneous nature of the samples. The XPS spectroscopic investigations allowed for identifying chemical bonds characteristic of the compounds constituting the main building blocks of kidney stones. The XPS studies showed also the presence of other compounds (mainly organic) on the surface of kidney stones which is consistent with the literature data.

XRD testing of sample 35 showed only the presence of calcium oxalates. Other studies revealed the presence of the additional components: phosphates and carbonates. A small amount of phosphorus determined by SEM-EDS may explain the lack of phosphates determined by XRD. Their amount was below the detection limit of the XRD method. The presence of phosphates was also confirmed by the XPS method. The absence of carbohydrates in the diffraction pattern is most likely due to the non-crystalline form of these compounds in the sample.

XRD testing of sample 37 showed only the presence of hydroxyapatite doped with minor amounts of carbonate and hydroxyls. These studies are consistent with the FTIR analysis. However, SEM-EDS and XPS studies showed the presence of phosphorus in the sample. In addition, the XPS spectrum made in a narrow range of binding energies for phosphorus showed the presence of PO_4_^3−^ bonds, which proves the presence of phosphates in the sample. As with the phosphate sample, it was below the detection limit of the XRD method.

XRD analysis of sample 43 showed only the presence of calcium oxalate monohydrate and hydrated calcium oxalate, hydroxyapatite doped with minor amounts of carbonate and hydroxyls, following a generic formula Ca_5_[(PO_4_)_2.823_ (CO_3_)_0.22_ (OH)_1.562_]. These studies are consistent with the FTIR analysis, SEM-EDS and XPS studies.

It is very important to combine different research methods for more insightful investigation and a detailed and in-depth understanding of the composition of urolithiasis helps to assess, treat and prevent the disease more effectively.

The research proved that all of the used research methods are effective tools for kidney stone identification. However, it should be noted that the use of more than one test method gives much more information about the studied material. It should be noted that one of the components of kidney stones is often carbonates, which co-precipitate in a non-crystalline form. Therefore, the use of only XRD X-ray diffraction will not identify this component of kidney stones. An additional method should be applied of identification of non-crystalline components of kidney stones, and these are spectroscopic methods.

## Figures and Tables

**Table 1:** Comparison of the obtained diffractogram of samples 35, 37 and 43 with the ICDD PDF4+2020 diffraction database.

Code	Name of Chemical Compound	Chemical Formula	Percantage Composition [%]
Sample 35			
04-009-6324	Calcium oxalate monohydrate	Ca(C_2_O_4_)(H_2_O)	100
Sample 37			
01-075-3729	Calcium hydroxyapatite doped with few carbonate and hydroxyls	Ca_5_[(PO_4_)_2.823_ (CO_3_)_0.22_ (OH)_1.562_]	100
Sample 43			
04-009-6324	Calcium oxalate monohydrate	Ca(C_2_O_4_)(H_2_O)	33
01-083-5351	Hydrated calcium oxalate	Ca(C_2_O_4_)(H_2_O)_2.35_	5
01-075-3729	Calcium hydroxyapatite doped with few carbonate and hydroxyls	Ca_5_[(PO_4_)_2.823_ (CO_3_)_0.22_ (OH)_1.562_]	62

**Table 2:** Surface elemental composition of sample 35.

Element	BE (eV)	% At. Conc.	% St. Dev.
C	284.9	73.9	3.15
N	399.9	2.2	2.43
O	531.9	18.9	1.58
Ca	347.4	2.8	0.92
P	133.4	0.8	1.33
Al	74.4	1.0	2.21
Na	1072.4	0.5	0.88

**Table 3:** Surface elemental composition of sample 37.

Element	BE (eV)	% At. Conc.	% St. Dev.
C	284.7	23.1	0.92
N	400.2	1.6	0.64
O	531.7	44.8	0.76
Na	1072.7	0.6	0.18
Mg	1305.2	0.7	0.24
P	133.2	13.2	0.36
Ca	347.2	15.9	0.30

**Table 4:** Surface elemental composition of sample 43.

Element	BE (eV)	% At. Conc.	% St. Dev.
C 1s	284.7	55.1	0.65
N 1s	399.7	2.7	0.61
O 1s	531.7	28.5	0.49
P	133.2	4.9	0.37
Ca	347.2	8.7	0.26

**Table 5:** Surface composition of carbon regions determined for samples 35, 37 and 43, BE—binding energy, FWHM—full width at half maximum, % At. Conc.—atomic concentration [41,42,43,44,45,46,47,48].

Sample Identifier	Name	BE (eV)	FWHM	% At. Conc.	Moieties
sample 35	C 1s	284.80	1.31	71.5	C-C/C-H
	C 1s	286.22	1.31	14.3	C-OH/C-O-C
	C 1s	287.70	1.31	5.2	C=O
	C 1s	288.70	1.31	7.4	COOH
	C 1s	289.51	1.31	1.7	carbonates
sample 37	C 1s	284.80	1.55	63.7	C-C/C-H
	C 1s	286.30	1.55	15	C-OH/C-O-C
	C 1s	287.60	1.55	5.5	C=O
	C 1s	288.60	1.55	12.3	COOH
	C 1s	289.84	1.55	3.5	carbonates
sample 43	C 1s	284.80	1.49	68.6	C-C/C-H
	C 1s	286.30	1.49	10.5	C-OH/C-O-C
	C 1s	287.60	1.49	4.6	C=O
	C 1s	288.60	1.49	13.9	COOH
	C 1s	289.46	1.49	2.4	carbonates

**Table 6:** Surface composition of oxygen regions determined for samples 35, 37 and 43, BE—binding energy, FWHM—full width at half maximum, % At. Conc. —atomic concentration [47].

Sample Identifier	Name	BE(eV)	FWHM	% At. Conc	Moieties
sample 35	O 1s	530.61	1.66	2.1	O^−2^/O=C
	O 1s	531.56	1.54	36.1	O=C-O-/O=P/CO_3_^−2^ (carbonates)
	O 1s	532.57	1.46	49.6	HO-C
	O 1s	533.63	1.32	9.7	HO-P
	O 1s	534.94	2.16	2.5	O_2_/H_2_O
sample 37	O 1s	530.83	1.67	6.1	O^−2^/O=C
	O 1s	531.37	1.59	76.3	O=C-O-/O=P/ CO_3_^−2^ (carbonates)
	O 1s	532.67	1.42	12.4	HO-C
	O 1s	533.66	1.35	3.8	HO-P
	O 1s	535.07	2.17	1.4	O_2_/H_2_O
sample 43	O 1s	530.77	1.50	6.2	O^−2^/O=C
	O 1s	531.36	1.59	45.4	O=C-O-/O=P/CO_3_^−2^ (carbonates)
	O 1s	532.28	1.52	36.2	HO-C
	O 1s	533.45	1.53	10.4	HO-P
	O 1s	534.76	1.85	1.7	O_2_/H_2_O

**Table 7:** Surface composition of calcium regions determined for samples 35, 37 and 43, BE—binding energy, FWHM—full width at half maximum, % At.—atomic concentration [47,48].

Sample Identifier	Name	BE (eV)	FWHM	% At Conc	Moieties
sample 35	Ca 2p 3/2	347.44	1.63	49.2	CaCO_3_/CaC_2_O_4_
	Ca 2p 1/2	351.01	1.81	50.8	CaHPO_4_
sample 37	Ca 2p 3/2	347.24	1.58	49.2	CaCO_3_/CaC_2_O_4_
	Ca 2p 1/2	350.81	1.72	50.8	CaHPO_4_
sample 43	Ca 2p 3/2	347.16	1.62	50.3	CaCO_3_/CaC_2_O_4_
	Ca 2p 1/2	350.72	1.70	49.7	CaHPO_4_

**Table 8:** Surface composition of phosphorus regions determined for samples 35, 37 and 43, BE—binding energy, FWHM—full width at half maximum, % At. Conc. —atomic concentration [47].

Sample Identifier	Name	BE (eV)	FWHM	% At. Conc.	Moieties
sample 35	P 2p 3/2	132.90	2.41	50.5	PO_4_^3−^
	P 2p 1/2	133.75	2.41	49.5	PO_4_^3−^
sample 37	P 2p 3/2	132.73	1.49	50.5	PO_4_^3−^
	P 2p 1/2	133.58	1.63	49.5	PO_4_^3−^
sample 43	P 2p 3/2	132.68	1.62	50.5	PO_4_^3−^
	P 2p 1/2	133.53	1.85	49.5	PO_4_^3−^

## Data Availability

The database is not available for direct access, but can be requested from researchers.

## Supplementary Materials

The following supporting information can be downloaded at: https://www.mdpi.com/article/10.3390/molecules28166089/s1, Figure S1. EDS-spectrograms of the sample 35; Figure S2. EDS-spectrograms of the sample 37; Figure S3. EDS-spectrograms of the sample 43.

## Sample Availability

Samples 35, 37 and 43 are available from the authors.
