# Supplementary material for: Chemical Studies of Multicomponent Kidney Stones Using the Modern Advanced Research Methods
Source: Molecules. 2023 Aug 16;28(16):6089. doi: 10.3390/molecules28166089 (PMC10458485; doi:10.3390/molecules28166089)
Supplement: Supplementary file 1 [file molecules-28-06089-s001.zip › molecules-2556992-supplementary.pdf]

*Supplementary Material*

# Chemical studies of multicomponent kidney stones using the modern advanced research methods

**Weronika Sofińska-Chmiel [1], Marta Goliszek [1], Marek Drewniak [1], Aldona Nowicka [1], Marcin Kuśmierz [1], Agnieszka Adamczuk [2], Paulina Malinowska [1], Ryszard Maciejewski [3,4], Małgorzata Tatarczak-Michalewska [5,*] and Eliza Blicharska [5,*]**

AREA A

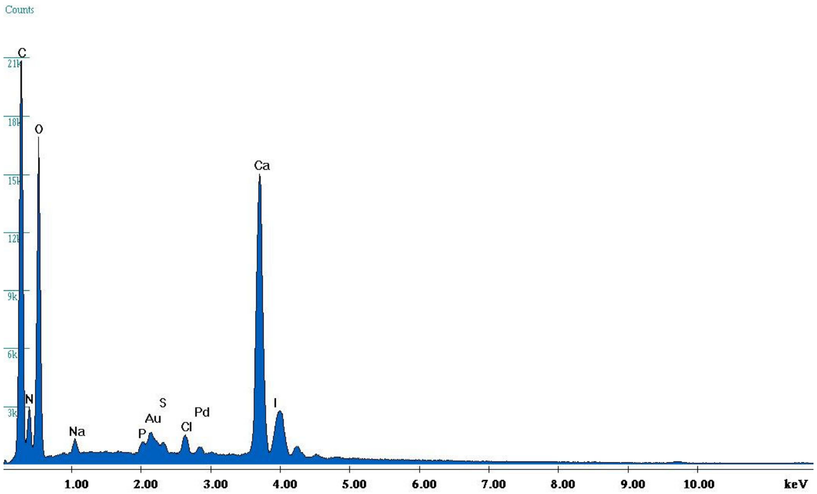


AREA B

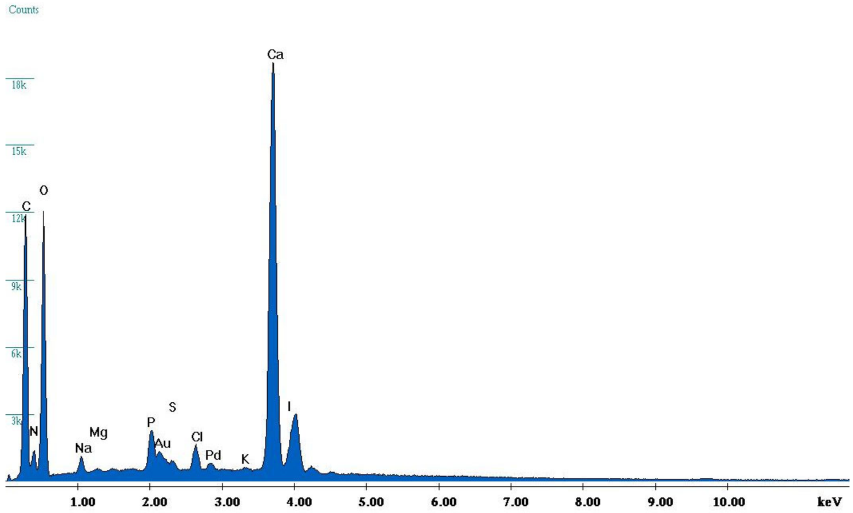

AREA C

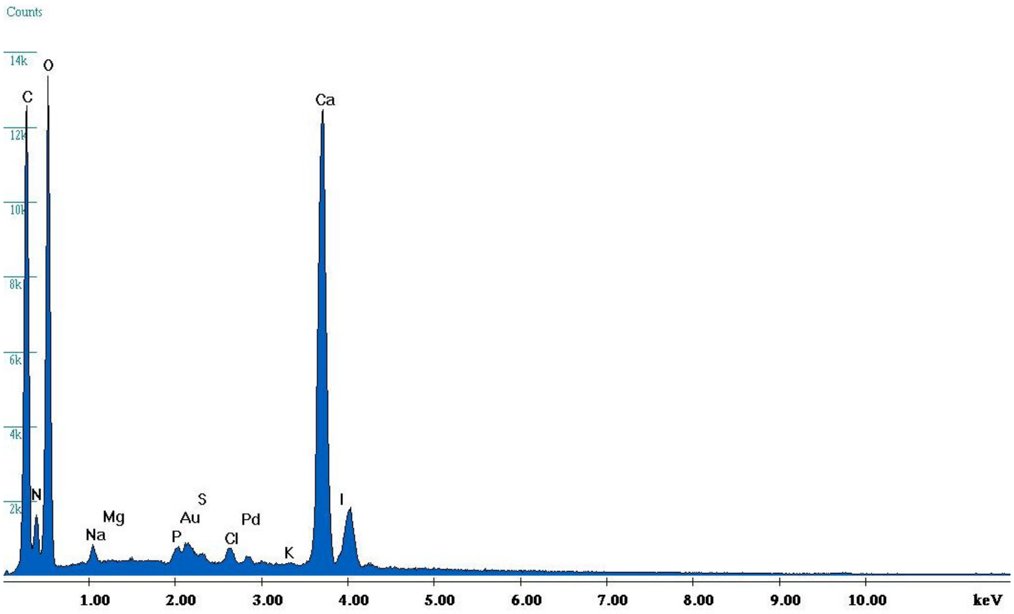


Figure S1. EDS-spectrograms of the sample 35.

AREA A

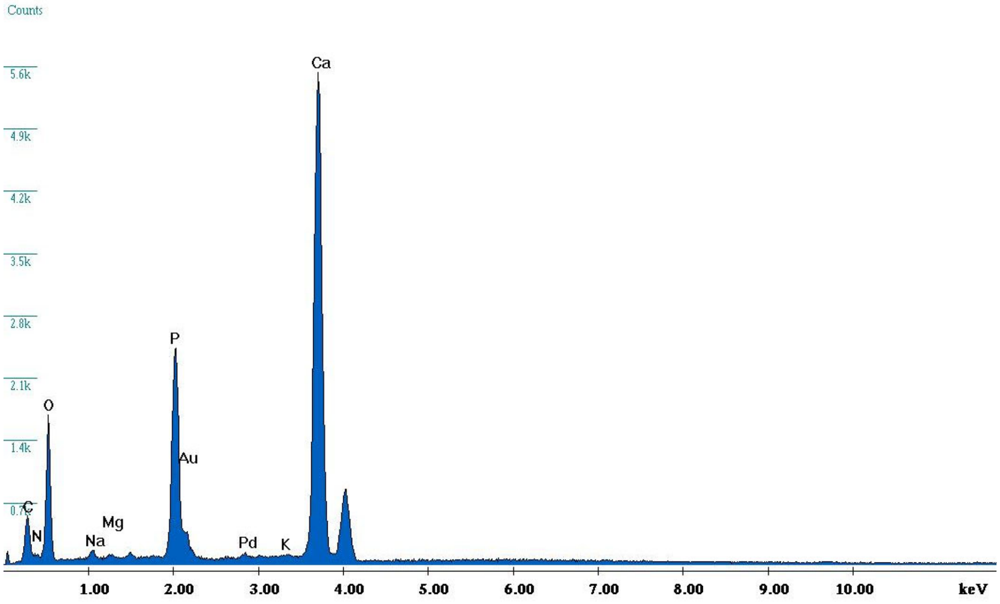


AREA B

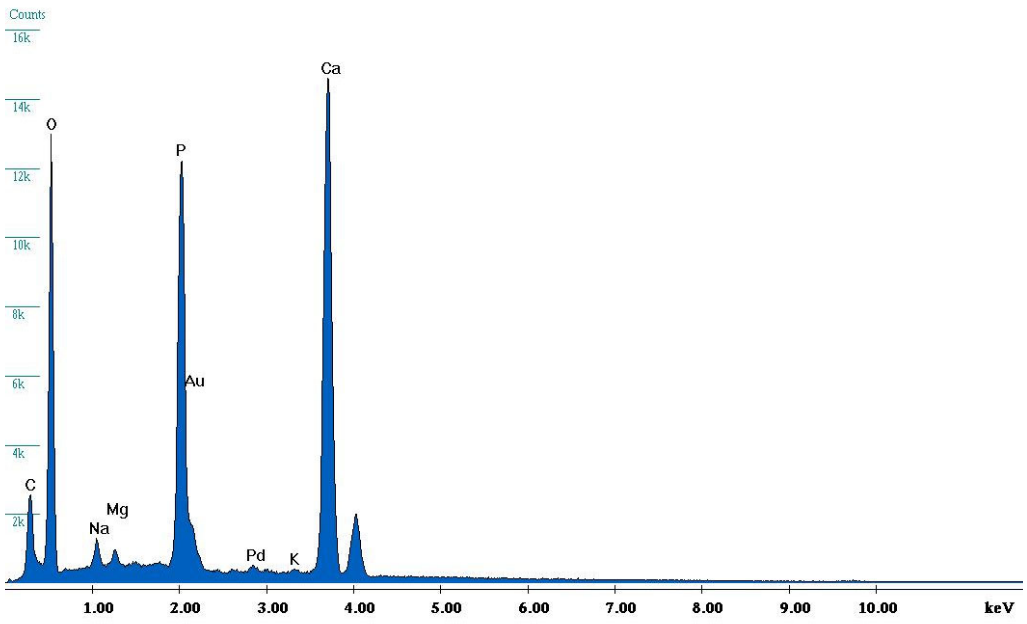


AREA C

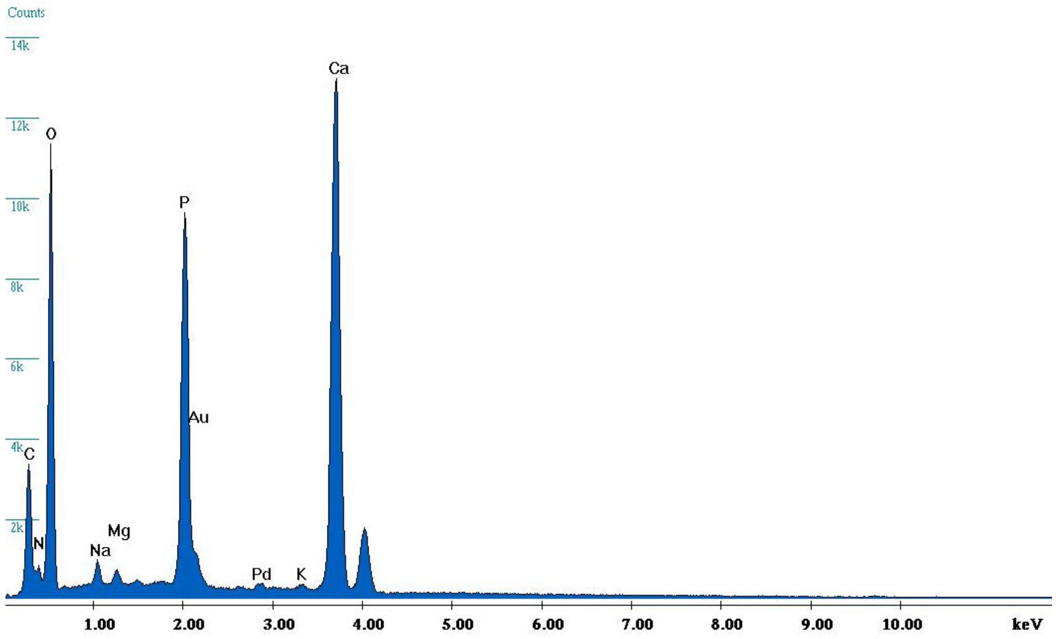


Figure S2. EDS-spectrograms of the sample 37.

AREA A

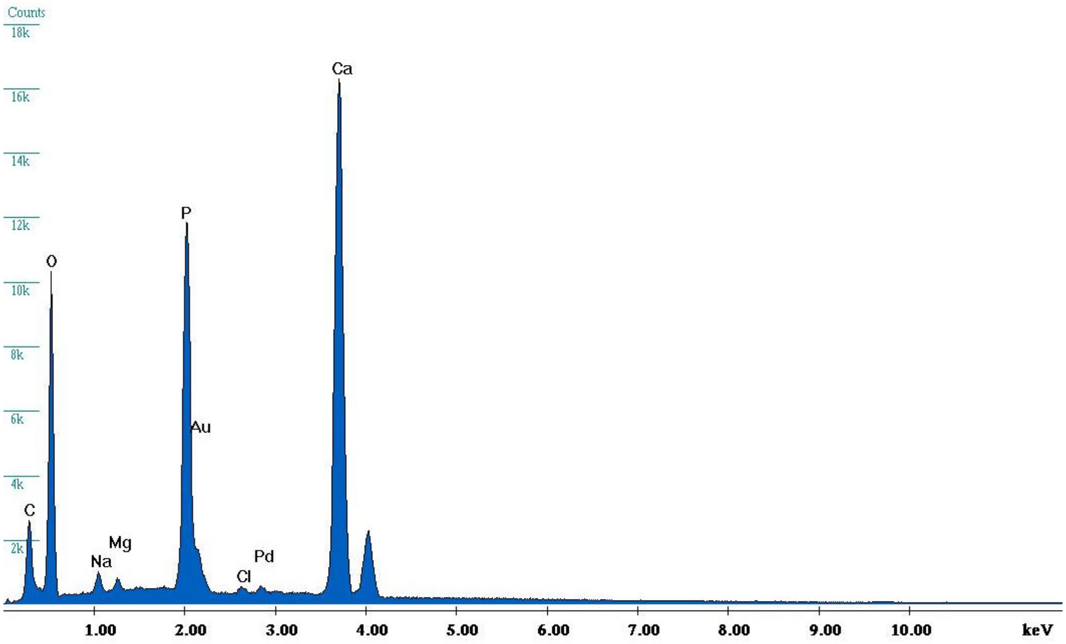
Counts
18k
16k
14k
12k
10k
8k
6k
4k
2k
Ca
P
O
Au
C
Mg
Na
Pd
Cl
1.00
2.00
3.00
4.00
5.00
6.00
7.00
8.00
9.00
10.00
keV

AREA B

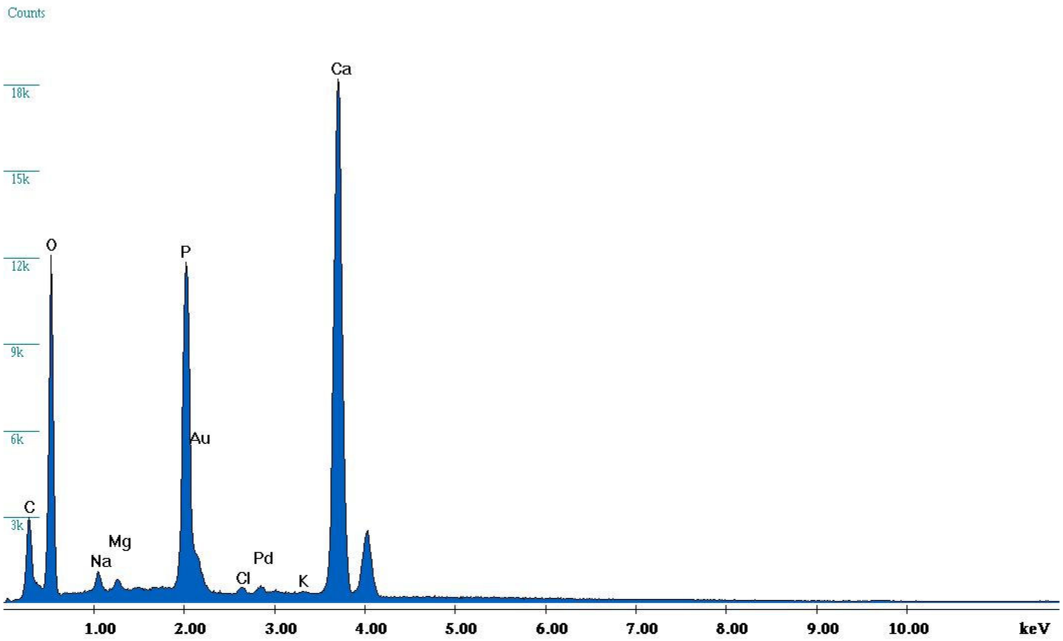
Counts
18k
15k
12k
9k
6k
3k
Ca
O
P
Au
C
Mg
Na
Pd
Cl
K
1.00
2.00
3.00
4.00
5.00
6.00
7.00
8.00
9.00
10.00
keV

AREA C

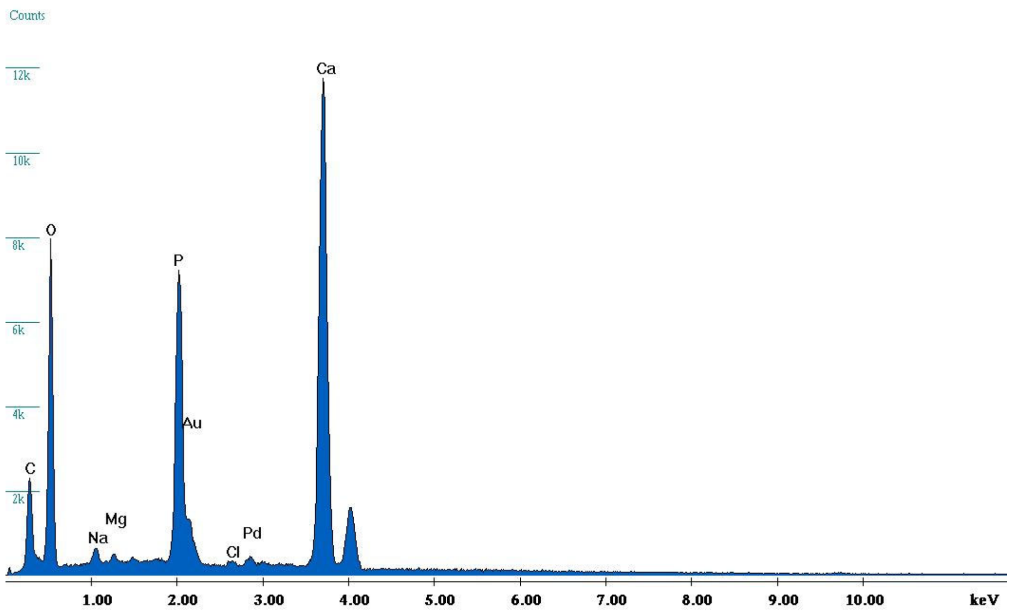


Figure S3. EDS-spectrograms of the sample 43.

The samples were sputtered with a Pd/Au target. These elements were not included in the process of generating the elemental composition using the software for the EDS Octane Elect Plus detector.
